# Alterations of several types of metabolites related to microbiota-gut-brain axis in urine of children with autism spectrum disorder

**DOI:** 10.3389/fpsyt.2026.1784658

**Published:** 2026-03-24

**Authors:** Kangwei Shen, Jingjing Huang, Dianzhi Liu, Qing Lu, Xuejun Kang, Yan Yan

**Affiliations:** 1 Key Laboratory of Child Development and Learning Science, Ministry of Education, School of Biological Sciences & Medical Engineering, Southeast University, Nanjing, China; 2 Changsha cultural creative and arts vocational college, Changsha, Hunan, China; 3 School of Education, Soochow University, Suzhou, China; 4 Laboratory of Environment and Biosafety Research Institute of Southeast University in Suzhou, Suzhou, China

**Keywords:** autism spectrum disorder, diagnosis, metabolomics, microbiota-gut-brain axis, urine

## Abstract

**Introduction:**

Multiple studies have shown that autism spectrum disorder (ASD) is accompanied by abnormalities in multiple metabolic pathways, however, the fluctuations of certain metabolites involved in these pathways have shown controversial results. This study aimed to identify metabolic characteristics that can distinguish children with ASD by using targeted metabolomics analysis on several types of metabolites related to the microbiota-gut-brain axis.

**Methods:**

A total of 54 children with ASD and 47 healthy children (HC) were recruited. Data from the Autism Behavior Checklist (ABC) scale for children with ASD were collected. Based on chromatography/mass spectrometry analysis, the metabolic characteristics of several types of metabolites related to the microbiota-gut-brain axis were discovered in urine samples. Single-variable and multi-variable analyses were conducted using MetaboAnalyst 6.0 and SPSS 27.0.1 to identify potential differential metabolites. The association between differential metabolite concentrations and ABC scores in children with ASD was evaluated using Spearman’s rank correlation analysis.

**Results and Discussion:**

Seven differential indicators, namely the ratio of cortisol to cortisone (R), creatinine, cortisol, taurine, histamine, homocysteine, and methionine were identified. The combined index diagnostic model constructed based on these indicators demonstrated strong discriminatory power, with an area under the receiver operating characteristic (ROC) curve of 0.943, a sensitivity of 92.6%, and a specificity of 93.7%. The above-mentioned biochemical indicators may be involved in the pathological physiological process of autistic behavioral symptoms from different aspects. These findings contribute to a better understanding of the underlying mechanisms of ASD.

## 1 Introduction

Autism spectrum disorder (ASD) is a neurodevelopmental disorder that starts in early infancy and young children, primarily characterized by social communication disorders, narrow interests or activities, repetitive behaviors, etc., which is one of the most important diseases leading to children’s disabilities (1). The pathogenesis of ASD is not fully understood, however, studies have shown that ASD and gastrointestinal dysfunction are co-existing (2). This may reflect abnormalities in bidirectional communication between the brain and the gut. The communication between the gut and the brain is known as the gut–brain axis, and the microbiota present in the gut can regulate brain function and produce behavioral phenotypes through the gut-brain axis by directly affecting the gut nervous system and producing bioactive metabolites in the gut (3–5). As a result, increasing interest is increasingly focusing on the potential impact of the microbiota-gut-brain axis on neurodevelopmental disorders. The hypothalamic-pituitary-adrenal (HPA) axis is another key mechanism in this bidirectional communication, and the gut microbiota is known to influence the endocrine system, such as affecting the HPA axis function. Under stress conditions, the HPA axis is over-activated, and the levels of circulating cortisol and pro-inflammatory cytokines are significantly increased, which can cause intestinal inflammation and changes in the composition of intestinal flora (6, 7).

Microbes in the human gastrointestinal tract produce many compounds that are useful to the host, including amino acids, certain B vitamins, and short-chain fatty acids (SCFAs) etc. (8, 9). For example, human gut bacteria can provide eight B vitamins, and at least four of them (B12, B9, B3, B6) cover more than a quarter of the recommended dietary intake (10). However, the results of studies on metabolites related to the microbial-gut-brain axis are inconsistent. For example, for cortisol, a representative marker of the HPA axis, has been reported to decrease in the morning plasma of children with ASD compared to healthy children (HC) (11–13). However, some research reported that there is no difference in cortisol levels between the two groups (14, 15). Variety of studies measured SCFAs in fecal samples of ASD children, some of them reported significantly increased level, some reported decreased level, while some studies reported non-significant changes between ASD and HC groups (14–17). Similar inconsistencies have been reported in the studies of amino acids in the urine of children with ASD. The authors suggest that the inconsistencies in the studies of these metabolic characteristics may be due to different populations, sample types, sample collection and sample preparation techniques, and data analysis techniques (18).

Numerous metabolomic studies have shown that ASD is accompanied by gut microbial metabolism, energy metabolism, oxidative stress, and other metabolic pathway diseases, but fluctuations in certain metabolites involved in these pathways have shown controversial results (19). For instance, a study on the urine of Italian children with autism has shown that there are disturbances in glucose metabolism among subjects with ASD, while C. Evans et al. reported non-significant differences in glucose excretion between ASD and control groups (20, 21). Metabolomics is divided into non-targeted metabolomics and targeted metabolomics. Non-targeted methods typically provide broader metabolite coverage than targeted methods but lack quantitative accuracy, showing lower reproducibility. Targeted metabolomics has significant advantages in quantification, and its measurement methodological parameters are more fully validated (22). This study adopted chromatography/mass spectrometry to conduct targeted metabolomics studies on several types of metabolites related to the microbial-gut-brain axis in urine, including amino acids, corticosteroids (cortisol, cortisone), SCFAs, and B vitamins (VB2, VB9, VB12), hoping to identify metabolic pathway abnormalities associated with autism and provide new ideas for the diagnosis and treatment of diseases.

## 2 Materials and methods

### 2.1 Subjects and samples

Fifty-four children with ASD (male: female = 47:7) aged 4–7 years were recruited from a special rehabilitation institution in Suzhou. A total of 47 children (boys: girls = 38:9) aged 5–6 years were recruited for HC group in two kindergartens in Suzhou. Children in the ASD group were diagnosed with ASD by professional psychologists and psychiatrists using relevant diagnostic tools before entering school. In order to investigate and evaluate the behavioral characteristics of children with ASD and further determine whether they meet the inclusion criteria, the guardian of the subjects was asked to fill in the Autism Behavior Checklist (ABC), which was compiled by KRUG in 1978, while collecting the samples of the subjects. There were 57 items in the scale, which were divided into five dimensions: sensory perception (S dimension), social interaction (R dimension), physical movement (B dimension), language (L dimension), and self-care (V dimension). The ABC questionnaires were distributed by a designated staff member and completed by parents or guardians in the consultation room under unified guidance. For each item, parents were instructed to indicate “yes” or “no.” The completed questionnaires were then collected promptly for analysis. The criteria for exclusion in the HC group were a history of mental illness, metabolic disease, or recent acute illness. All the children had not taken any drugs in the last 1 month, to rule out the endocrine interference of the drugs. The experiment met the ethical requirements of the World Health Organization on the use of human subjects, and was approved by the Ethics Committee of Zhongda Hospital affiliated to Southeast University. Before the experiment, the experimenter fully explained the purpose and process of the experiment to the guardian, as well as the impact on the subject, and sent the guardian a written informed consent. The guardians of the subjects were informed that they could withdraw from the study at any time. After receiving the consent, the subjects’ guardians signed an informed consent form.

First-morning urine samples were collected from subjects with ASD upon waking. On school days, the subjects were awakened by their parents/guardians at about 8:00 AM and arrived at the special school or kindergarten by 9:00 AM. Guardians were instructed to collect the first morning urine sample using pre-issued sterile disposable urine cups. Immediately after the collection, the container was sealed and brought to the kindergarten on the same day. Upon receipt, the researchers immediately transferred the urine samples into sterile centrifuge tubes and stored them at -20 °C until analysis.

### 2.2 Data analysis

The ABC scale was collected, and the scores of ASD children in the five dimensions of sensory perception (S dimension), social interaction (R dimension), physical movement (B dimension), language (L dimension), and self-care (V dimension), and the total score of the scale (T) were analyzed. The correlation between metabolite levels and scale scores in ASD group was evaluated using Spearman’s correlation. A p-value less than 0.05 was considered statistically significant.

The urinary concentrations of metabolites were adjusted by urinary creatinine concentration to control the influence of the fluctuation in urine volume. The concentration of the analytes was present in μg/mg Cr. All target compounds were naturally Ln-transformed for drawing diagrams using GraphPad Prism 10.1.2. Statistical analysis was performed using IBM SPSS 27.0.1, and descriptive statistics of creatinine-adjusted urinary target compounds levels in children were performed using Mann-Whitney test to compare the differences between the groups with and without ASD.

The metabolomics dataset was processed using MetaboAnalyst v6.0. All missing values were replaced using the mean of each variable within the group and natural logarithmic transformations of the variables were performed. Principal component analysis (PCA) and orthogonal partial least squares discriminant analysis (OPLS-DA) were performed to visualize metabolic changes between children with HC and ASD.

### 2.3 Metabolites analysis

#### 2.3.1 Determination of urinary creatinine

Before testing, the samples were naturally thawed at room temperature, then the urine sample was centrifuged at 12000 rpm for 5 min, and the supernatant was ready for use. Creatinine levels were determined using the method specified in the Chinese National Standard (WS/T 98-1996). The high-performance liquid chromatography (HPLC) system comprised an SPD-10AD UV detector (Shimadzu Corporation, Japan) with a Shimadzu LC-20AD, operating at a flow rate of 0.9 mL/min and at ambient column temperature. The sample injection volume was set to 20 μL, with UV detection at a wavelength of 254 nm. For analyte separation, a Shimadzu VP-ODS C18 chromatographic column (5μm, 250 mm × 4.6 mm) was used. Chromatographic separation was accomplished using a mobile phase consisting of 95% aqueous solution (0.05 mol/L sodium acetate) and 5% methanol.

#### 2.3.2 Urinary cortisol and cortisone analysis

According to the methods reported in the literature for the determination of cortisol and cortisone in urine, solid phase extraction (SPE) columns packed with polystyrene (PS) nanofibers were used to extract the target substances in urine. To 500 μL of urine supernatant, D4-cortisol was added as the Internal Standard (IS), reaching a concentration of 20 ng/mL. The mixture was added to the PS nanofibers column, and the liquid was slowly pressed out drop by drop with a barometric solid phase extractor, so that the target compound was adsorbed on the nanofibers material. Then 100 µL of methanol was added and eluted in the same way. 10 µL of eluent was injected into liquid chromatography-mass spectrometry for determination, and the instrument setting was shown in reference (23).

#### 2.3.3 Urinary B vitamins analysis

Determination of three B vitamins (VB2, VB9, VB12) in urine with reference to the literature method (24). SPE columns filled with 5 mg of polystyrene/polypyrrole (PS/PPy) nanofibers were used for sample processing. 50 µL of 2 mg/mL diphenylboronic acid 2-Aminoethylester (DPBA) solution was added to 500 µL of urine supernatant. After vortex mixing, the mixture was added to the PS/PPy nanofibers column, and the liquid was slowly pressed out drop by drop with a barometric solid phase extractor, so that the target compounds were adsorbed on the nanofibers material. Before elution, the PPy column was eluted with 2 mg/mL of DPBA solution for three times (100 µL/time), and then eluted with 100 µL eluent (20 µL formic acid mixed with 80 µL mobile phase). The eluent was collected and vortexed, and 20 µL was injected into an HPLC system for detection, and the instrument setting is shown in reference.

#### 2.3.4 Urinary SCFAs analysis

Based on the SCFAs analytic method established by our research group (25). Sample pretreatment was accomplished by a packed–fiber solid phase extraction method using 5 mg of PS/PPy nanofibers as the sorbent. Before solid phase extraction, the nanofibers in the extraction column were activated with 200 μL of methanol and 200 μL of water, respectively. The 500 μL of urine supernatant was pushed through the nanofibers using a barometric solid phase extractor, and the target compounds will remain on the adsorbent. Then the column was eluted with 100 μL of 0.01 mmol/L hydrochloric acid ethanol solution. The collected eluent was vortexed, and 1μL of it was injected into GC-MS for detection. Other instruments and experimental conditions remained unchanged.

#### 2.3.5 Urinary amino acid analysis

After thawing and centrifuging the urine, 500 µL of the urine sample was passed through a PS nanofiber column for purification. The eluate was collected, mixed, and analyzed according to the method described in reference (26). The mass spectrometer used was a 3200 QTRP (Applied Biosystems, USA), and positive ion detection mode was employed using an electrospray ionization source. The spray voltage was set at 450.0 V, the collision pressure at 0.1995 Pa, the sheath gas and auxiliary gas settings at 206.85 kPa and 68.95 kPa, respectively, and the ion source temperature at 350 °C. The analysis was conducted in the selective reaction monitoring (SRM) mode with a scan time of 0.5 s.

## 3 Results

### 3.1 General characteristics

The average age of the ASD group was 4.5 ± 1.4 years, while that of the HC group was 5.6 ± 0.5 years. In the ASD group, 87.0% were boys and 13.0% were girls, while in the HC group, 87.2% were boys and 12.8% were girls. The scale scores indicate that the average total score of the ABC scale was 85.5 ± 14.9, among which the average score of sensory perception (ABC_S), social interaction (ABC_R), physical movement (ABC_B), language (ABC_L) and self-care (ABC_V) were 16.8 ± 4.5, 19.7 ± 3.8, 16.8 ± 4.6, 18.2 ± 5.1, and 14.3 ± 4.2, respectively. **Table 1** shows the demographic characteristics of 54 children with ASD and 47 healthy children, as well as the scale scores of the ASD group.

**Table 1:** Demographic characteristics of the study population and scale scores in ASD group.

Profile of the participants			ASD	TD	*P* value
Characteristics	No		54	47	
	Sex	Male	47	41	0.506
		Female	7	6	
	Age (years)	Mean	4.5 ± 1.4	5.6 ± 0.5	<0.001
Assessment scales of autism behavior checklist (ABC)	Sensory perception (ABC_S)		16.8 ± 4.5	–	
	Social interaction (ABC_R)		19.7 ± 3.8	–	
	Physical movement (ABC_B)		16.8 ± 4.6	–	
	Language (ABC_L)		18.2 ± 5.1	–	
	Self-care (ABC_V)		14.3 ± 4.2	–	
	Total score		85.5 ± 14.9	–	

ASD, autism spectrum disorder; ABC, autism behavior checklist; HC, healthy children. *P* values are calculated using the Chi-square test for sex and the Mann–Whitney U test for age.

### 3.2 Urine metabolite levels in children with ASD and HC

**Figure 1:**
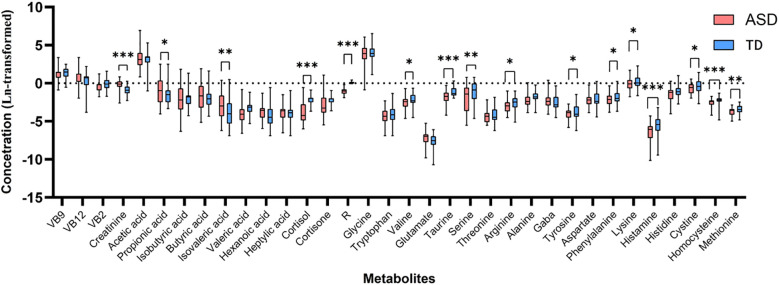
Univariate analysis of metabolite concentrations of ASD groups and HC group. (Boxplots illustrate the upper quartile, median (central transverse line), and lower quartile, with whiskers indicating the maximum and minimum values. The asterisk indicates a significant difference: *p <0.05; **p <0.01; ***p<0.001 compared with HC group).

As shown in **Table 1**, there was a significant difference in the ages of the two groups of children. Taking the ASD/HC grouping as the fixed factor, the concentration of each metabolite as the dependent variable, and age as the covariate, a one-way analysis of covariance was conducted. The results showed that age only had a significant main effect on the level of hexanoic acid (F = 5.088, *p* < 0.05), and had no significant effect on the levels of the other metabolites (all *p* > 0.05). After correcting for the influence of age through covariance analysis, urine metabolite levels in children with ASD and HC are shown in **Figure 1**. The ratio of cortisol to cortisone (R) was also included as an indicator, because it can represent the 11β-HSD1 enzyme that can convert the inactive cortisone into the active cortisol. 11β-HSD1 enzyme can exert anti-inflammatory or pro-inflammatory effects in metabolic-related inflammatory diseases and immunity by regulating the synthesis of glucocorticoids.

There were statistical differences between the levels of multiple metabolites in the urine of children in the ASD group and the HC group, among which the levels of creatinine, propionic acid, and isovaleric acid were significantly increased in the ASD group. More metabolites were significantly reduced in the ASD group, including cortisol, R, valine, taurine, serine, arginine, tyrosine, phenylalanine, lysine, histamine, homocysteine, and methionine.

### 3.3 Multivariate analysis and identification of potential biomarkers

**Figure 2:**
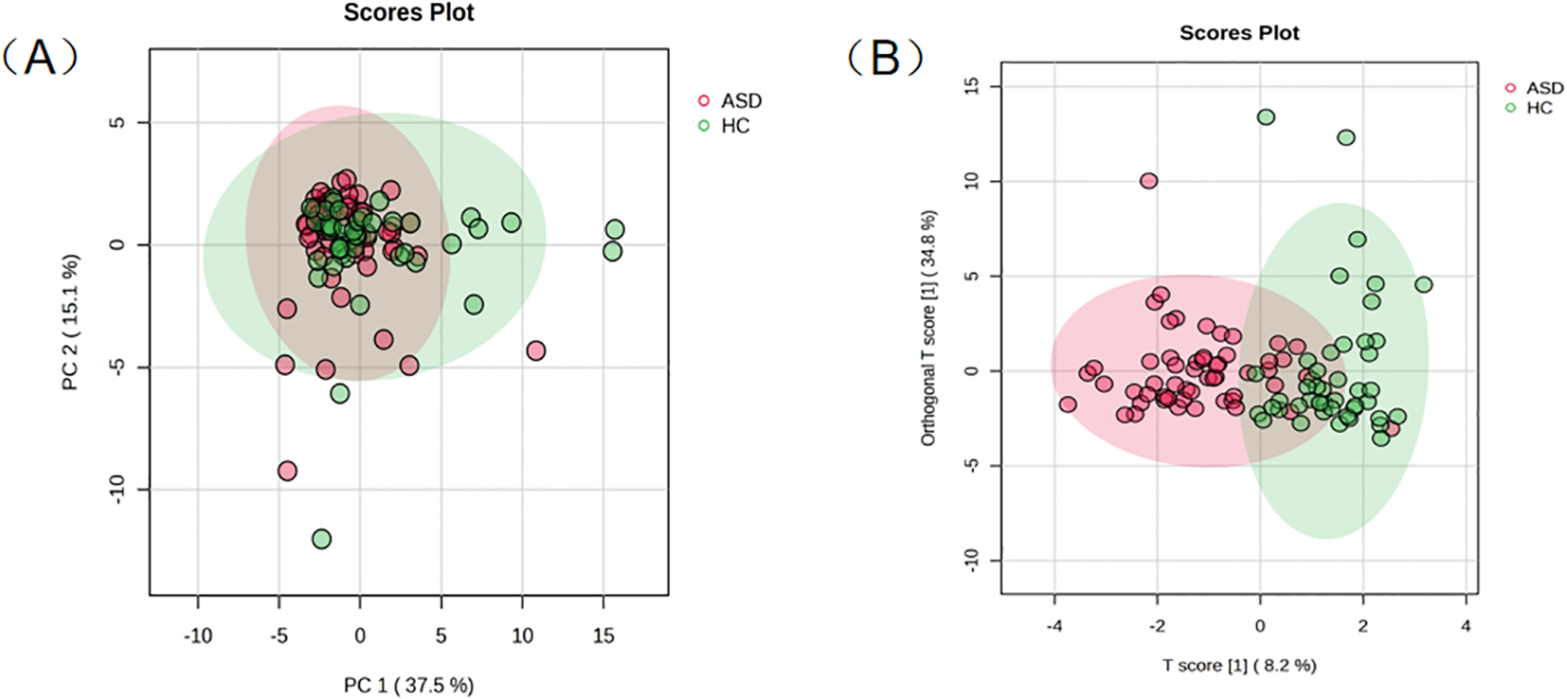
Identification of differential metabolites in the urine of ASD and HC. **(A)** The PCA between ASD and HC groups; **(B)** OPLS-DA score plots based on the urine samples from ASD and HC groups.

To identify the differential metabolites between ASD and HC group, various statistical methods were employed for the corresponding experiments. First, the confounding effect of age was eliminated through linear regression. Regression models were constructed for each of the 34 metabolites with ASD/HC group and age as independent variables, and the residuals were extracted for subsequent statistical analysis. An unsupervised PCA was used to generate an overview of variations between groups. **Figure 2A** shows the PC1 vs. PC2 score plot for all the samples, however, the PCA distribution did not identify a valid separation between the HC and ASD groups. Then, an orthogonal partial least squares discriminant analysis (OPLS-DA) model was attempted to be established. The OPLS-DA score plots of urinary metabolite profiling among the HC and ASD groups showed that the two groups could be discriminated weakly by metabolite profiling with the model fitness and predictability (R2Y = 0.636, and Q2 = 0.387) after 100-fold permutation tests (**Figure 2B**).

**Figure 3:**
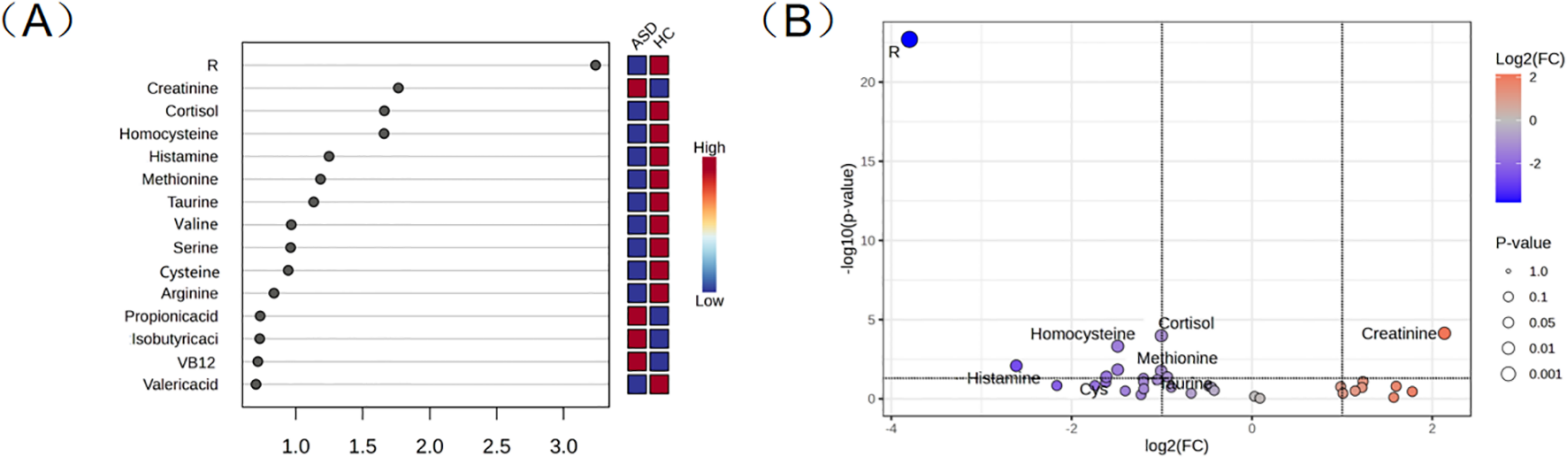
**(A)** Important features identified by the OPLS-DA. **(B)** Volcanic map of metabolites.

According to OPLS-DA, the variable importance in the projection (VIP) values of each metabolite in sample classification can be calculated. **Figure 3A** shows the VIP scores of 15 metabolites, among which 8 metabolites with VIP > 1 are included. Among them, 7 metabolites were down-regulated and 1 metabolite was up-regulated in the ASD group. The metabolic differences between the two groups were visualized and data mined by drawing volcano maps. As shown in **Figures 3B**, using |log2FC| ≥1 and P < 0.05 as screening criteria, a total of seven differential metabolites were identified in the ASD vs HC group, namely R, creatinine, cortisol, taurine, histamine, homocysteine, and methionine.

**Figure 4:**
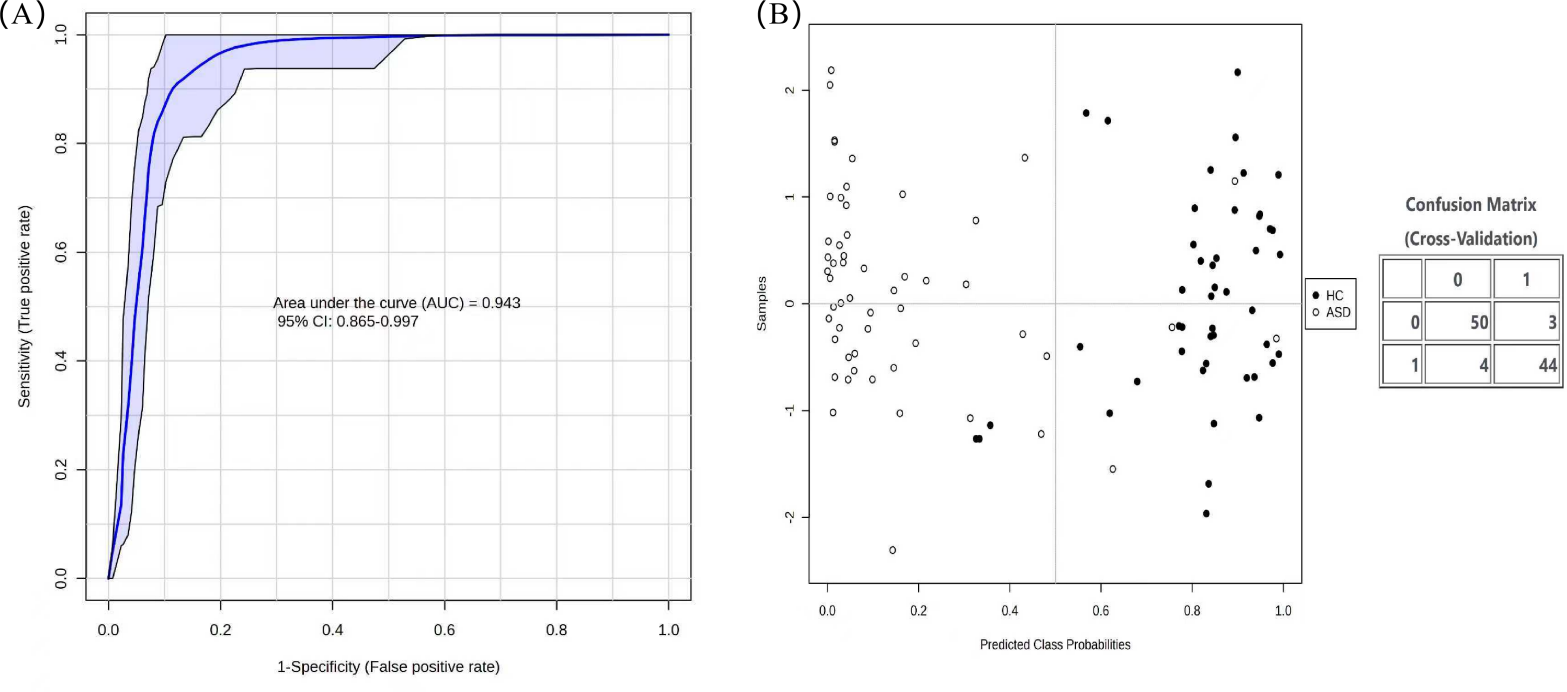
Indentification of potential diagnostic biomarkers. **(A)** Combined ROC curve; **(B)** prediction probability of R, cortisol, creatinine, taurine, histamine, homocysteine, and methionine.

Seven differential metabolites, R, creatinine, cortisol, taurine, histamine, homocysteine, and methionine were analyzed using MetaboAnalyst 6.0 for biomarkers, and multivariate ROC analysis was performed for the seven metabolites. A metabolite combination prediction model was constructed based on the Support Vector Machine (SVM) model. The area under the ROC curve (AUC) of this SVM model was 0.943, with a 95% confidence interval of 0.855 to 0.997. Permutation test revealed a significant difference (*P* < 0.01), indicating the model was robust. Under the optimal cut-off value, the model’s sensitivity was 92.6% (50/54) and the specificity was 93.7% (44/47), and the average accuracy based on 100 cross validations was 88.9% (**Figure 4**).

### 3.4 Analysis of correlation between urinary metabolites and ABC scale scores

**Figure 5:**
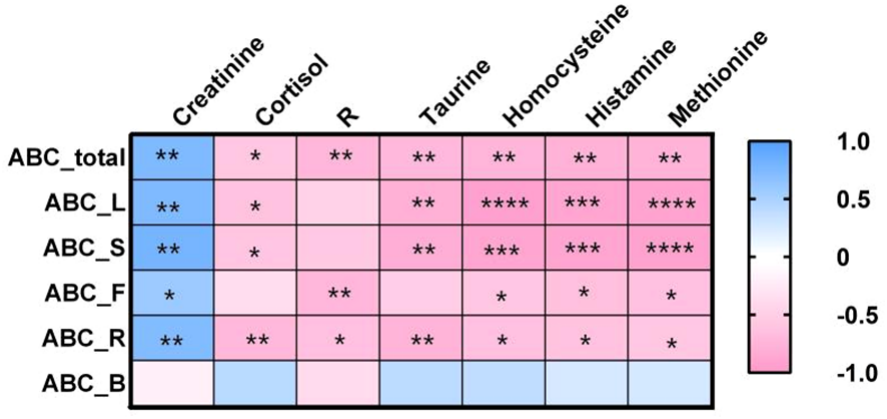
Correlation map calculated using Spearman correlation coefficient between the score of ABC scale and seven differential metabolites in urine. (Spearman correlation coefficient is reported with colour gradient, blue color indicates positive association, while pink indicates negative association. The figure in the lattice represents a significant correlation: *p <0.05; **p <0.01; ***p <0.001; ****p <0.0001).

After correcting the age, the residuals of the seven differential metabolites were extracted. Spearman correlation analysis was then conducted to examine the correlation between the post-correction metabolite residuals and the ABC scale scores. The results are shown in **Figure 5**. The results showed that scores of ABC_total, ABC_L, ABC_S, ABC_F, and ABC_R were significantly negatively correlated with cortisol, R, taurine, homocysteine, histamine, and methionine (*p* < 0.05 to *p* < 0.0001). however, positive correlations were observed between these scores and creatinine. The relationship between physical movement (ABC_B) score and the seven indicators had an opposite correlation pattern, however, none of them reached significance.

## 4 Discussion

In view of the inconsistent research results regarding the metabolic characteristics of the microbiota-brain-gut axis in ASD children, we collected morning urine samples to avoid the influence of diet, and adopted the packed-nanofiber solid phase extraction created by our research team to purify and treat biological samples. The characteristics of several types of metabolites related to microbial-brain-gut axis in children with ASD were studied using targeted metabolomics analysis with good quantitative accuracy and repeatability. We observed some changes in five types of metabolites, namely B vitamins, SCFAs, glucocorticoids, amino acids, and creatinine, in urine of children with ASD. Univariate analysis found that 16 of 34 metabolites showed significant differences between the ASD and HC groups. Using individual metabolite datasets, an OPLS-DA model was established to visualize the metabolic alteration patterns between the ASD and HC groups. With the seven selected differential indicators, namely the ratio of cortisol to cortisone (R), creatinine, cortisol, taurine, histamine, homocysteine, and methionine, ASD cases could be distinguished from HC group, with an area under the receiver operating characteristic curve of 0.943, a sensitivity of 92.6%, and a specificity of 93.7%. Given that this study is an exploratory metabolomics research with a relatively small sample size, the presented model represents preliminary results. In the future, its clinical translational potential and universality can be further confirmed by expanding the sample size and conducting multi-center external cohort validations.

Three types of urinary B vitamins, VB2, VB9, and VB12 were measured in this work. The concentrations of VB9 and VB2 in ASD group were lower, while the concentrations of VB12 were higher than those in HC group (although not significant). Most studies have reported that children with ASD lack B vitamins. Li et al. studied the relationship between serum folic acid (VB9) and VB12 levels with intestinal flora. It was found that the levels of serum VB9 and VB12 in ASD group were lower than those in HC group, and the serum levels of the two B vitamins are correlated with the abundance of some intestinal flora (27).

However, no significant differences in plasma VB9 and VB12 between ASD and HC groups have also been reported (28). Furthermore, studies have shown that serum levels of VB12 are higher in patients with ASD than in HC group (29). Recently, Zhang et al. inferred the genetic role of serum homocysteine and B vitamins levels on autism spectrum disorders through Mendelian randomization, and found that elevated serum VB12 levels may increase the risk of ASD, but such significant associations have not been found for serum levels of VB6, VB9, and homocysteine. The authors note that if these findings are confirmed in subsequent studies, it suggests that serum VB12 levels in children with ASD are specific and that excess VB12 may have toxic effects (30).

The two vitamins, VB9 and VB12 play a role in amino acid metabolism, such as cysteine, methionine, and homocysteine conversion. Studies have shown a negative correlation between serum homocysteine, VB9, and VB12 levels in children with ASD (26). This negative correlation was also supported by the fact that urinary homocysteine concentrations in ASD patients tested in this study were significantly lower than those in the HC group. In addition, the literature also indicates that VB12 is involved in SCFAs (propionic acid) metabolism and is also involved in the regulation of cortisol (31). The significantly decreased urinary cortisol and propionic acid in the ASD group were also observed in this study, which indicated that the abnormal VB12 level in ASD patients was closely related to the abnormal metabolism of cortisol and propionic acid.

Most studies on SCFAs reported in the literature measured fecal samples, and increases, decreases, and no change in fecal SCFAs for ASD patients have been reported, reflecting inconsistent findings (14–17). The conflicting data may be explained by Boets’s point of view that colon SCFAs production and changes cannot be characterized by fecal measurements because colon cells absorb SCFAs quickly, resulting in only approximately 5% of SCFAs being excreted in feces. In addition, massive metabolism of SCFAs in colon cells and liver results in low plasma SCFAs levels (32).

In this study, urine SCFAs were measured, although the concentration of SCFAs in the urine was also very low. We utilized nanofiber-based solid-phase extraction developed by our research group to concentrate SCFAs in urine, reducing the interference signals and improving the detection sensitivity of them in urine. It was found that in the urine of children with ASD, the concentration of isovaleric acid and propionic acid were significantly increased, while the changes of the other six SCFAs were not significant. Animal studies have shown that intraventricular injection of propionate into young mice causes autism-like behavioral and physiological changes and Clostridia spp. is known to produce exotoxins and propionate, resulting in an inflammatory state that may exacerbate autism symptoms (33, 34). These studies support the idea that urine propionic acid may play an important role in the development of autism.

Studies on the HPA axis in children with ASD mainly used saliva and plasma samples for measurement, while urine samples were less frequently used. The results of this study showed that the levels of cortisol in the urine samples of children with ASD were lower than those in HC group, which was similar to the results from plasma sample studies reported in the literature (14, 15). It was found that the ratio of urinary cortisol to cortisone (R) was significantly decreased. The urinary R value can be used to assess the activity of 11β-steroid dehydrogenase (especially 11β-HSD2) (35). At the cellular level, cortisol and cortisone can be converted into each other under the action of 11β-steroid dehydrogenase. 11β-steroid dehydrogenase has two subtypes: type I (11β-HSD1) and type II (11β-HSD2). The function of 11β-HSD2 is to convert biologically active cortisol into inactive cortisone, while 11β-HSD1 has the opposite effect and can compensate for the decrease in cortisol caused by its conversion into cortisone (36). Therefore, the results of this experiment reflect the imbalance in the antagonistic effect between 11β-HSD1 and 11β-HSD2. Compared with HC group, the creatinine level in the urine of children with ASD was also significantly increased. Given that the increase in urine creatinine levels is related to the decline in renal excretion function, it suggests that the HPA axis and renal function may be abnormal in children with ASD.

Amino acids are of vital importance in brain function. They not only serve as metabolic intermediates and materials for protein synthesis, but also act as mediators for neural communication. However, the changes in amino acids in the body fluids of ASD patients have inconsistent results reported in the literature (37). Chen et al. have demonstrated that elevated levels of plasma neuroactive amino acids (glutamate) and decreased levels of essential amino acids are major characteristics of plasma amino acids in children with autism (38). The results of this study on urine also showed similar findings, namely that the level of glutamate in urine increased (but not reach a significant level), while the concentrations of certain essential amino acids in urine, such as lysine, methionine, phenylalanine, and valine decreased significantly. Threonine, histidine, and tryptophan also decreased but did not reach a significant level. Histidine is a semi-essential amino acid for children. Its metabolite, histamine, is one of the most important participants in several physiological and pathological processes (including immune regulation function, control of gastrointestinal secretion, neurotransmitters in the central nervous system, and regulation of vascular permeability) (39). We found that the concentrations of urinary histamine in children with autism were significantly lower than those in the HC group. We also found that several sulfur-containing amino acids in the urine of children with ASD were decreased, including taurine, methionine, cysteine, and homocysteine (among which cysteine did not reach a significant level). These amino acids are involved in one-carbon metabolism, and the results supported the abnormality of various physiological processes in children with ASD, including nucleotide synthesis, amino acid homeostasis, epigenetic maintenance, and redox balance etc. (40).

To explore the relationship between behavioral abnormalities and biochemical indexes in children with ASD, we analyzed the correlation between seven differential metabolites and the total score (T) of the children’s autism Behavior Checklist (ABC scale) and the score of five dimensions, including sensory perception, social interaction, physical movement, language and self-care. The results showed that the total score of ABC was significantly negatively correlated with R, cortisol, taurine, methionine, histamine, and homocysteine, and positively correlated with creatinine. The correlations of the subscales of sensation, social interaction, language, and self-care with these indicators were similar, with only creatinine showing a positive correlation, while the others were all negatively correlated. The subscale of physical movement showed opposite correlations with the seven indicators, although not reaching significance. This suggests that the imbalance of excitation-inhibition, abnormal methylation metabolism, and dysfunction of the hypothalamic-pituitary-adrenal axis may jointly participate in the occurrence and development of different behavioral symptoms of autism. Creatinine, histamine, homocysteine, taurine, methionine, cortisol, and 11β-HSD can be potential biological markers for evaluating the severity and phenotypic characteristics of autism behavioral symptoms.

However, this study also has some other limitations. Firstly, the sample size is relatively small, with only 54 children with ASD and 47 healthy children included. This relatively small sample size may limit the general applicability of the research results and increase the possibility of selection bias. Repeating these results in a larger cohort would make the conclusions more convincing. Another limitation is that information on potential confounding factors (such as diet, weight, and medication) was not examined. These factors can affect the metabolic profile and may introduce additional variability in the results. Another issue is the use of urine creatinine to normalize the levels of metabolites. However, subsequent analysis revealed that creatinine was also a differential metabolite, and the differences in its levels may cause deviations in the standardization process and affect the bias of statistical analysis. If other normalization strategies were used for comparison simultaneously, the results may be more reliable. Furthermore, our participants were preschool children from one city in China. Therefore, the research conclusions may not be applicable to all individuals with ASD in different regions, races, and ages.

## 5 Conclusions

This study examined the metabolic characteristics of children with ASD related to the microbiota-gut-brain axis and compared them with the metabolic characteristics of healthy children. The results revealed significant changes in the levels of some metabolites. Four differential indicators, namely R, creatinine, cortisol, taurine, histamine, homocysteine, and methionine, were identified as the potential biomarkers for ASD children. A prediction model containing these seven parameters was developed, which may be helpful in improving the diagnostic level of ASD. The study also explored the correlations between the seven identified differential metabolites and the scores in children’s autism Behavior Checklist. Overall, this study provides valuable insights into the metabolic changes related to ASD and helps in exploring the underlying mechanisms for intervention and treatment of the disease.

## Data Availability

The raw data supporting the conclusions of this article will be made available by the authors, without undue reservation.
